# miR-148a regulates expression of the transferrin receptor 1 in hepatocellular carcinoma

**DOI:** 10.1038/s41598-018-35947-7

**Published:** 2019-02-06

**Authors:** Kamesh R. Babu, Martina U. Muckenthaler

**Affiliations:** 1 Department of Pediatric Hematology, Oncology, and Immunology, University of Heidelberg, Heidelberg, Germany; 2 Molecular Medicine Partnership Unit, University of Heidelberg, Heidelberg, Germany

## Abstract

Transferrin receptor 1 (TFR1) is a transmembrane glycoprotein that allows for transferrin-bound iron uptake in mammalian cells. It is overexpressed in various cancers to satisfy the high iron demand of fast proliferating cells. Here we show that in hepatocellular carcinoma (HCC) TFR1 expression is regulated by miR-148a. Within the TFR1 3′UTR we identified and experimentally validated two evolutionarily conserved miRNA response elements (MREs) for miR-148/152 family members, including miR-148a. Interestingly, analyses of RNA sequencing data from patients with liver hepatocellular carcinoma (LIHC) revealed a significant inverse correlation of TFR1 mRNA levels and miR-148a. In addition, TFR1 mRNA levels were significantly increased in the tumor compared to matched normal healthy tissue, while miR-148a levels are decreased. Functional analysis demonstrated post-transcriptional regulation of TFR1 by miR-148a in HCC cells as well as decreased HCC cell proliferation upon either miR-148a overexpression or TFR1 knockdown. We hypothesize that decreased expression of miR-148a in HCC may elevate transferrin-bound iron uptake, increasing cellular iron levels and cell proliferation.

## Introduction

MicroRNAs (miRNAs) are a class of evolutionary conserved short non-coding RNAs (~22nt) that regulate gene expression at the post-transcriptional level by binding to miRNA response elements (MREs)^1^, sites with partial complementarity within the 3′ untranslated region (3′UTR) of target messenger RNA (mRNA). Binding of miRNAs to MREs causes mRNA cleavage and degradation^2^ or translational repression^3^, depending on the extent of miRNA:mRNA base pairing complementarity. miRNA expression is dysregulated in human cancers and frequently associated with cancer prognosis^4^. Specifically, miR-148a, a member of the miR-148/152 family, is downregulated in several cancer subtypes including breast cancer^5^, gastric cancer^6^, colorectal cancer^7^, pancreatic cancer^8^, hepatocellular carcinoma (HCC)^9,10^, esophagus cancer^11^, non-small cell lung cancer^12^, and prostate cancer^13^. Moreover, decreased miR-148a expression in tumors is frequently associated with an advanced clinical stage, metastasis, and poor survival^14^.

**Figure 1:**
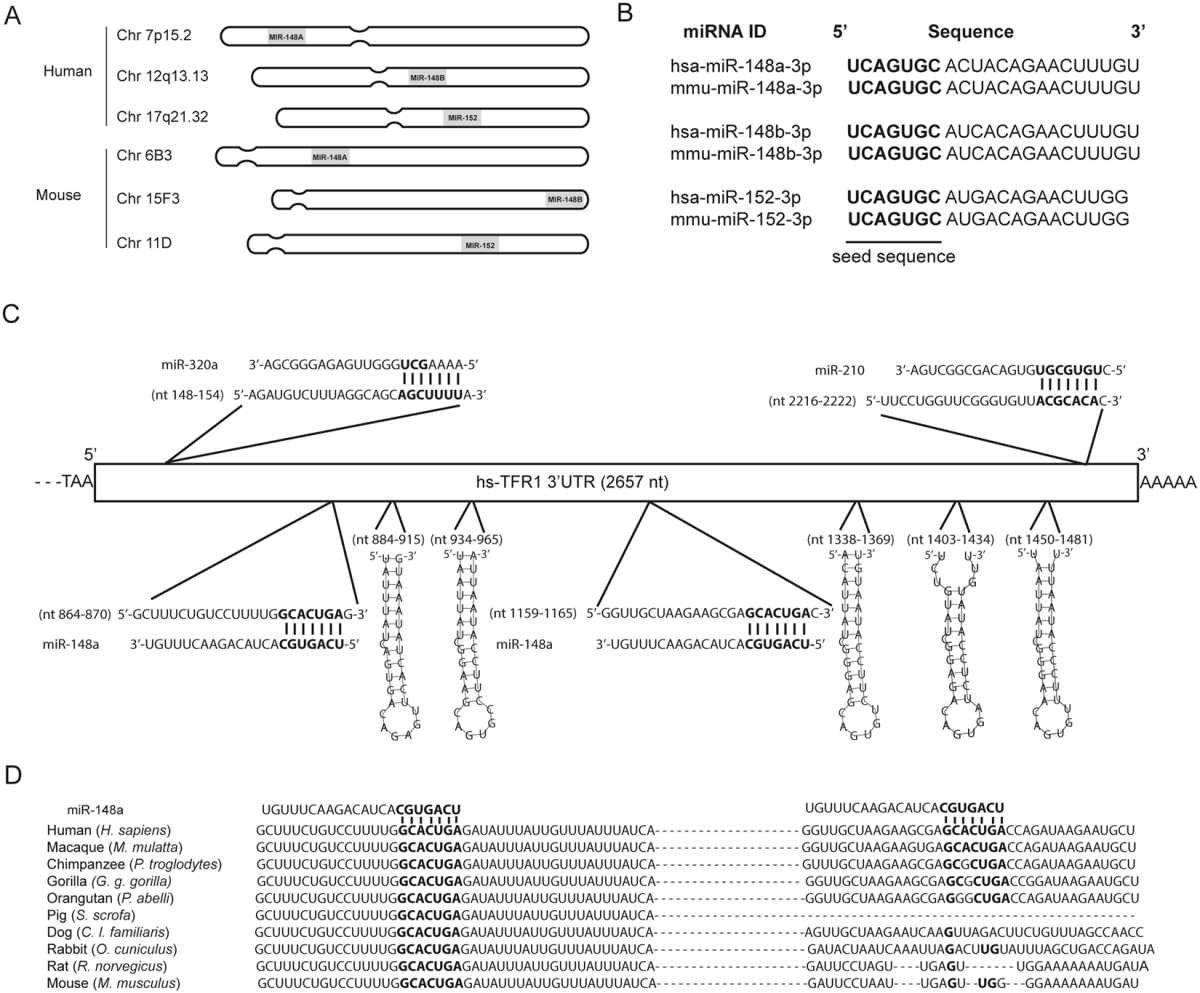
The TFR1–3′UTR contains highly conserved miRNA response elements (MREs) for miR-148a. (**A**) Chromosomal location of the miRNA members of the human and mouse miR-148/152 family. (**B**) Human and mouse miR-148/152 family members show highly conserved seed sequences (bold). (**C**) Location of miRNA response elements (MREs) for miR-320a, miR-148a and miR-210 (bold), and five iron-responsive elements (IREs) (stem-loop) in the human TFR1–3′UTR. (**D**) Sequence alignment of the miR-148a seed sequence and its binding site (bold) in the TFR1–3′UTR of ten mammalian species.

The miR-148/152 family consists of three highly conserved miRNA members: miR-148a, miR-148b and miR-152, which are located on human chromosome 7, 12 and 17, and on mouse chromosome 6, 15 and 11, respectively^15^ (Fig. 1A). Despite miR-148/152 expression from different chromosomal loci in human and mouse, the mature miRNAs are similar and share conserved seed sequences (Fig. 1B). Suppression of miR-148a expression in tumors occur at the level of transcription^16–18^ and methylation^19–21^. Downregulation of miR-148a contributes to cancer pathogenesis, as miR-148a regulates genes associated with cell proliferation, apoptosis, metastasis and invasion (as reviewed in^14^). Among miR-148a target genes are those that play a role in cell growth and proliferation, such as hematopoietic PBX-interacting protein (HPIP)^17^, insulin receptor substrate 1(IRS-1)^5^, insulin-like growth factor-1 receptor (IGF-IR)^5^, receptor tyrosine-protein kinase erbB3 (ERBB3)^22^ and mitogen-inducible gene-6 (MIG6)^23^, during the cell cycle, such as cullin related protein (CAND1)^24^, M-phase inducer phosphatase 2 (CDC25B)^25^ and the DNA methyltransferase 1 (DNMT1)^26^, as well as the anti-apoptotic protein B-cell lymphoma 2 gene (BCL-2)^27^.

Iron (Fe) is an essential nutrient required for numerous cellular functions, including cell growth and proliferation. It is required for DNA synthesis as a co-factor of the ribonucleotide reductase^28^, as well as the regulation of proteins associated with cell cycle control such as GADD45, p21 and p53^29,30^. Iron is essential for cellular growth and proliferation signaling pathways such as JAK-STAT3^31^, mammalian target of rapamycin (mTOR)^32^, and Wnt signaling^33^. Cellular iron availability is regulated by a network of genes that control cellular iron uptake, storage, utilization and export^34^. An increasing number of studies reported that genes associated with iron metabolism are regulated by miRNAs under physiological and pathophysiological conditions^35–38^ as well as in cancer^39–41^. Furthermore, in many cancer subtypes including HCC, systemic and intracellular iron homeostasis is altered^42,43^. Especially, abnormal iron uptake^44^ and hepatic iron overload^43^ is observed in HCC patients.

Transferrin receptor 1 (TFR1) is a broadly expressed transmembrane protein best known for its function in transferrin-bound iron (Tf-Fe) uptake in most cell types, including cancer cells^45^. One report additionally suggests a role in the uptake of iron-bound ferritin^46^. Furthermore, it is also involved in intracellular signaling. Binding of either polymeric A1 isotype immunoglobulins (pIgA1) or Tf-Fe to TFR1 in erythroblasts increases sensitivity to erythropoietin (Epo) by activating mitogen-activated protein kinase (MAPK) or phosphatidylinositol 3-kinase (PI3K) signaling pathways^47^. Destearoylation of TFR1 activates c-Jun N-terminal kinase (JNK) signaling, leading to E3 ubiquitin-protein ligase HUWE1-dependent mitofusins (MFN) ubiquitination, attenuated MFN activity and mitochondrial fragmentation^48^. TFR1 expression is regulated post-transcriptionally by the iron regulatory proteins (IRPs) and tristetraprolin (TTP) that bind to five iron responsive elements (IREs)^34^ and AU-rich elements (ARE)^49^ at its 3′UTR, respectively. TFR1 transcription is activated by the hypoxia inducible factor (HIF) in response to hypoxia^50^ and iron deficiency^51^, by Stat5 binding to GAS sequences in its first intron^52^ as well as by the oncogenic transcription factor c-Myc^53^. In addition, TFR1 expression is controlled post-translationally by CD133^54^, epidermal growth factor receptor (EGFR)^55^ and c-Abl kinase^56^. TFR1 is overexpressed in various cancer subtypes^57^, including breast cancer^58^, esophageal squamous cell carcinoma^59^, HCC^60^, oral cancer^61^ and pancreatic cancer^62^.

Here we show that the TFR1 3′UTR encompasses evolutionarily conserved MREs for miR-148a, a member of the miR-148/152 family. Analysis of TCGA-RNA sequencing datasets of patients with liver hepatocellular carcinoma (LIHC) revealed that the expression of TFR1 is significantly increased in the tumor correlating with decreased miR-148a expression. Ectopic expression of miR-148a in the HCC cell lines HepG2 and Huh7 significantly downregulates TFR1 expression post-transcriptionally and decreases proliferation of HCC cells.

## Materials and Methods

### Cell culture

HepG2 and Huh7 cells were purchased from ATCC (Cat. No. HB-8065) and Cell Lines Service (Cat. No. 300156), respectively. Cells were cultured in DMEM medium (Invitrogen, Cat. No. 11965-092) supplemented with 10% FBS (Invitrogen, Cat. No. 10500064). Cell cultures were maintained at 37 °C under 5% CO_2_.

### RNA extraction, reverse transcription and quantitative real-time PCR

RNA extraction, reverse transcription and quantitative real-time PCR (qPCR) were performed as previously described^40^. Briefly, total RNA as well as miRNA was extracted from hepatocellular carcinoma cells using miRNeasy Micro Kit (Qiagen, Cat. No. 217084), according to manufacturer’s protocol. mRNAs and miRNAs were reverse transcribed using the RevertAid RT Reverse Transcription kit (Thermo Scientific, Cat. No. K1691) and miScript II RT kit (Qiagen, Cat. No. 218161), respectively. Quantification of mRNA and miRNA were performed using SYBR Green Master Mix (Applied Biosystems, Cat. No. 4309155) and miScript SYBR-Green PCR kit (Qiagen, Cat. No. 218073), respectively. QPCR was performed using the ABI StepOne Plus Real-Time PCR system (Applied Biosystems, Cat. No. 4376600). Relative expression levels of mRNA and miRNA was calculated using the ΔΔCt method^63^. Sequence of primers used are listed in the Table S1.

### Cloning and site-directed mutagenesis

The protein coding sequence (CDS) of human TFR1 was amplified from the cDNA of HepG2 cells by PCR and cloned into the BamH1-XbaI restriction sites of the pcDNA Mammalian expression vector (Invitrogen, Cat. No. V79020). The complete 3′UTR sequence of the human DNMT1 and TFR1 genes were amplified from genomic DNA of HepG2 cells by PCR. 3′UTR of DNMT1 and TFR1 were cloned into the SacI-NheI restriction sites of the pmirGLO Dual-Luciferase miRNA Target Expression Vector (Promega, Cat. No. E1330) to generate pMIR-DNMT1 and pMIR-TFR1, respectively. To generate pMIR-RPL19 construct, a double stranded DNA oligonucleotide encompassing the complete 3′UTR of human RPL19 was directly cloned into the vector. In addition, a double stranded DNA oligo nucleotide with the identical sequence of miR-148a was cloned into the pmirGLO vector in the sense (+) or antisense (−) orientation to generate a positive (pMIR-148a^+^) or negative (pMIR-148a^−^) control vector, respectively. Sequences of primers used are listed in the Table S2. The QuickChange II XL Site-Directed Mutagenesis Kit (Agilent Technologies, Cat. No. 200522) was used to generate mutations in the predicted miR-148a response elements within the 3′UTR of DNMT1 and TFR1. Sequence of primers used are listed in Table S3. All the constructs were verified by plasmid DNA sequencing.

### Transfection of siRNAs, miRNA mimics/Inhibitors and plasmids

TFR1 short interfering RNAs (siRNAs) (Ambion, Cat. No. 4390824) and miR-148a mimic (Ambion, Cat. No. 4464066), were transfected using the Lipofectamine RNAiMAX reagent (Invitrogen, Cat. No. 13778150), whereas plasmid vectors were transfected using the Lipofectamine 2000 reagent (Invitrogen, Cat. No. 11668019).

### Dual-luciferase reporter assay

Dual-luciferase reporter assays were performed as previously described^40^. HepG2 cells (5 × 10^3^ cells/well) were seeded into a sterile 96-well white assay plate (Corning, Cat. No. CLS3610-48EA). After 24 h, 50 nM of miR-148a mimic or negative control (NC) (Ambion, Cat. No. 4464058) were transfected into cells using RNAiMAX reagent. Twenty-four hours later, cells were transfected with 10 ng of pMIRGLO luciferase constructs using Lipofectamine 2000 reagent. 24 h post-transfection, cells were lysed with 1X passive lysis buffer (Promega, Cat. No. E1941). Luciferase activity was analyzed using the Dual Luciferase Reporter assay system (Promega, Cat. No. E1960) and the Centro LB 960 luminometer (Berthold Technologies, Cat. No. 38100-50).

### Cell growth curve analysis

At 24 h post-transfection, cells were trypsinized and plated (5 × 10^3^ cells/well) into a sterile 96-well culture plate with complete growth medium (Corning, Cat. No. CLS3595). 24 h later, medium was aspirated, and cells were washed with PBS. 50 µL of 0.5% crystal violet (Sigma, Cat. No. V5265) was added to each well and incubated for 20 min at ambient temperature. Wells were washed with H_2_O and air-dried for 2 h at room temperature. 200 µL of methanol (Sigma, Cat. No. 322415) was added to each well and incubated for 20 min at room temperature on a bench rocker. Optical density of each well was measured at 570 nm using the Spectramax M Series microplate reader (Molecular devices). Optical density was determined at the interval of 24 h for 5 days.

### Soft agar assay

A 0.6% agarose base was prepared in sterile 6-well culture plates. At 24 h post-transfection, HepG2 and Huh7 cells were trypsinized and plated (10 × 10^3^ cells/well). Cells were mixed with complete growth medium and agarose to a final concentration of 0.3%. Cell mixture was plated above the base followed by incubation at 37 °C with 5% CO_2_. Cells were supplemented with fresh complete growth medium every two days. After 10 days, the cell colonies were imaged under 0.5X magnification (Olympus SZX12) and quantified using ImageJ v.1.51k.

### Western blot analysis

Cells were harvested and washed twice with ice-cold PBS (Sigma, Cat. No. 806552). Cells were lysed with RIPA buffer supplemented with protease inhibitors (Roche, Cat. No. 11697498001). Protein was quantified using the DC protein assay (BioRad, Cat. No. 5000111). Protein samples were resolved by 10% SDS-PAGE and transferred to nitrocellulose membrane (GE Healthcare, Cat. No. 10600002). Primary antibodies directed against TFR1 (ThermoFisher, Cat.No. 13-6800) and ACTB (Sigma, Cat.No. SAB2100037) were used. Western blot image capture and densitometric analysis were performed using the Fusion-Fx system (Vilber Lourmat).

### *In silico* miRNA target prediction and TCGA RNA-seq datasets

Correlation of TFR1 and miRNA expression across various human tissues and cells was generated using mimiRNA (http://mimirna.centenary.org.au/mep/formulaire.html)^64^. Potential miRNAs that target TFR1 3′UTR were predicted using nhmmer sequence alignment tool^65^ and miRNA target prediction algorithms: Targetscan v.7.0^66^, PicTar^67^ and RNA22 v.2.0^68^. TCGA RNA-seq datasets of Liver Hepatocellular carcinoma (LIHC) was downloaded from the UCSC Xena browser (http://xena.ucsc.edu/)^69^.

### Statistical analysis

Statistical analysis were performed using Prism v.6 (GraphPad). Data were represented as mean ± SEM of at least three independent experiments. Pearson’s correlation coefficient was applied to analyze the correlation between expression levels of miR-148a and TFR1. To determine the overall survival of the LIHC cohorts, Kaplan-Meier analyses was used and the Mantel-Cox test was applied to evaluate the significance. Two-tailed Student’s *t* test was applied to determine the significance for luciferase assay, qRT-PCR data and densitometric analysis. Two-way ANOVA was employed to evaluate statistical difference between the growth curves of the cells. *P* < 0.05 were considered statistically significant.

## Results

### The TFR1-3′UTR contains highly conserved MREs for miR-148/152 family members

To determine whether the TFR1 3′UTR contains MREs, we examined the human TFR1 3′UTR (2657nt) for sequence complementary to 7 nucleotide seed sequences of miRNAs by using the nhmmer sequence alignment tool^65^. We selected those MREs with high evolutionary conservation and that were additionally predicted by the following miRNA target prediction algorithms: Targetscan v.7.0^66^, PicTar^67^ and RNA22 v.2.0^68^. We identified two putative MREs for members of the miR-148/152 family, which consists of 3 miRNA members (miR-148a, miR-148b and miR-152). Despite the fact that each miRNA member is located on a different chromosome (Fig. 1A), they encompass conserved seed sequences in human and mouse (Fig. 1B). For follow up analysis, we chose miR-148a as a representative of the miR-148/152 family. The two MREs for miR-148a are located at nt864–870 and nt1159–1165 within the human TFR1 3′UTR (Fig. 1C). The human TFR1 3′UTR contains additional experimentally validated MREs for miR-320a and miR-210 that are located upstream and downstream of the miR-148a MREs, respectively (Fig. 1C)^38,70^. Furthermore, it also encompasses five iron-responsive elements (IREs). The first two IREs are located between the two miR-148a MREs and the other three are located downstream of the second miR-148a MRE (Fig. 1C). Importantly, the two miR-148a MREs show high evolutionary conservation among mammals but not in other vertebrates, whereby the MRE located at nt864–870 is conserved across ten mammalian species (Fig. 1D). Our bioinformatics analyses suggest that the miR-148/152 family may target the TFR1 3′UTR.

### TFR1 is targeted by miR-148a

**Figure 2:**
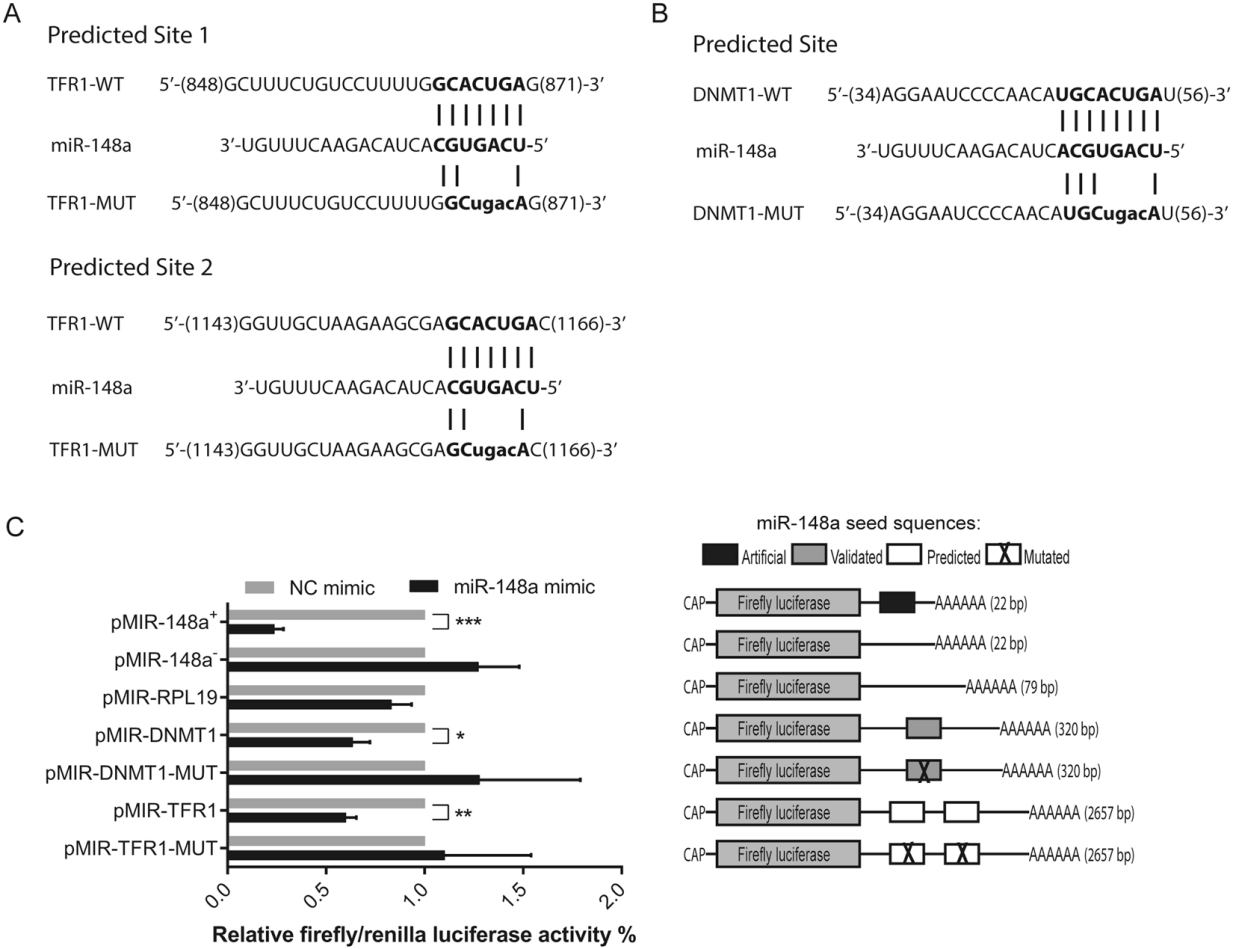
TFR1 is a direct target for miR-148a. HepG2 cells were transfected with 50 nM of miR-148a mimic or a negative control (NC). At 24 h post-transfection, the luciferase reporter constructs pMIR-148a^+^ (sequence complementary to miR-148a), pMIR-148a^−^ (sequence identical to miR-148a), pMIR-RPL19, pMIR-DNMT1, pMIR-TFR1, (**A**) pMIR-TFR1-MUT (mutated miR-148a response element; bold and lower case) or (**B**) pMIR-DNMT1-MUT (mutated miR-148a response elements; bold and lower case) were transfected. (**C**) Luciferase activity was measured 48 h later. Experiments were performed in triplicates and repeated at least three times. Data are represented as mean ± SEM, and the values from the negative control (NC) was set to 100% *P < 0.05, **P < 0.01, ***P < 0.001, 2-tailed Student’s t test.

To analyze whether miR-148a directly and specifically targets TFR1 via the predicted miR-148a response elements in the 3′UTR, we created luciferase reporter constructs containing either the entire 3′UTR sequence of human TFR1 (referred herein as pMIR-TFR1) or a derivative where the miR-148a response elements were mutated (Fig. 2A). As a negative control the complete 3′UTR of the human RPL19 gene was cloned into the reporter construct (pMIR-RPL19), lacking the predicted miR-148a response elements. As a positive control we inserted the complete 3′UTR of the human DNMT1 gene (pMIR-DNMT1), a validated miR-148a target, as well as a mutant derivative (Fig. 2B)^26^. Furthermore, artificial positive and negative control constructs with perfect sequence complimentary to miR-148a or identical to miR-148a (pMIR-148a^+^ and pMIR-148a^−^, respectively) were analyzed. HepG2 human hepatocarcinoma cells were transfected with miR-148a mimics and luciferase reporter constructs. miR-148a overexpression strongly reduced luciferase activity from cells transfected with the positive control constructs pMIR-148a^+^ and pMIR-DNMT1 whereas the luciferase activity was unaltered in those cells transfected with the negative control constructs pMIR-148a^−^, pMIR-RPL19 or pMIR-DNMT1-MUT (Fig. 2C), indicating that miR-148a overexpression is efficient and specific. Moreover, miR-148a overexpression has significantly reduced luciferase activity of pMIR-TFR1 but not of pMIR-TFR1-MUT, in which the predicted miR-148a responsive elements were mutated (Fig. 2C). Altogether, these data show that miR-148a directly targets the TFR1–3′UTR.

### TFR1 mRNA expression is inversely correlated to miR-148a expression in hepatocellular carcinoma

**Figure 3:**
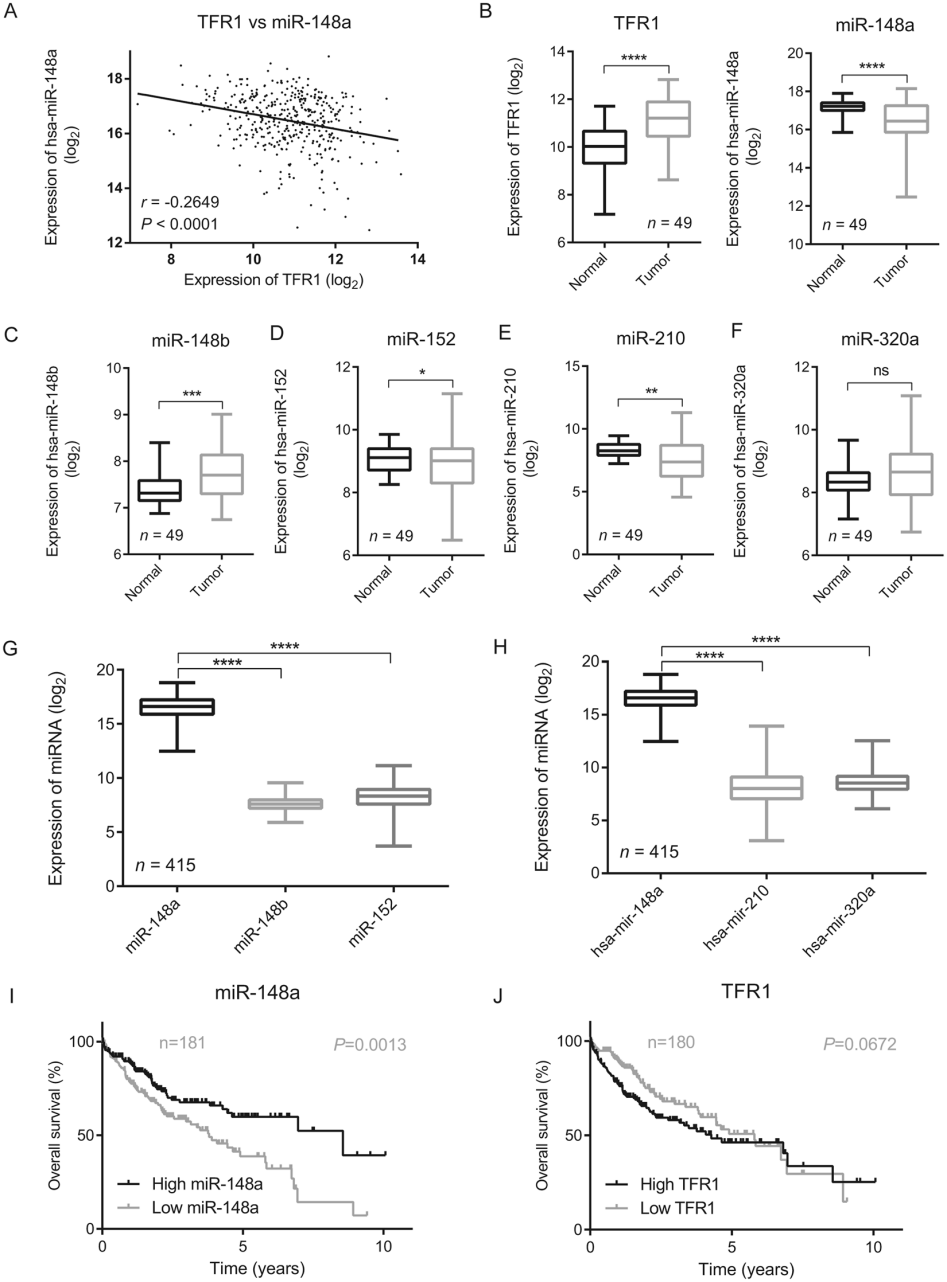
TFR1 mRNA expression is inversely correlated to the expression of miR-148a in HCC. TCGA RNA-sequencing dataset of hepatocellular carcinoma (HCC) patients was downloaded from the UCSC Xena browser (http://xena.ucsc.edu/)^69^. (**A**) Scatter plot shows negative correlation between miR-148a and TFR1 mRNA expression in HCC patients (*n* = 419, *r* = −0.2649, *P* < 0.0001), Pearson’s correlation coefficient was used. (**B**) The boxplot demonstrates that in the tumor of HCC patients miR-148a levels are significantly decreased (*n* = 48, *P* < 0.0001) while TFR1 mRNA expression is significantly increased (*n* = 49, *P* < 0.0001) compared to matched normal healthy tissues. (**C**–**H**) Boxplots illustrating expression levels of the indicated miRNA members in the tumor and matched normal healthy tissue within TCGA dataset of HCC patients, ns = no statistical significance, *P < 0.05, **P < 0.01, ***P < 0.001, ****P < 0.0001, 2-tailed Student’s t test. Kaplan-Meier plot illustrates better survival among HCC patients with (**I**) higher miR-148a expression levels (*n* = 181, *P* = 0.0013) and a trend towards better survival with (**J**) lower TFR1 mRNA expression levels (*n* = 180, *P* = 0.0672), Mantel-Cox test was applied.

We next examined the clinical importance of the interactions between miR-148a, a miR-148a/152 family member whose expression is frequently altered in cancer^71^ and TFR1 by analyzing RNA expression data from cancer patients. We restricted our analysis to RNA sequencing data sets accessible within the Cancer Genome Atlas (TCGA) (http://cancergenome.nih.gov/) to differentiate expression levels of each miRNA member of the miR-148/152 family. We observed that expression of TFR1 mRNA is significantly negatively correlated to miR-148a expression (*P* < 0.001) in patients with hepatocellular carcinoma (HCC) (*n* = 419) (Fig. 3A). Moreover, we found that in HCC patients expression of miR-148a was significantly reduced in the tumor (*P* < 0.001) compared to adjacent normal tissue (Fig. 3B). Conversely, TFR1 mRNA expression was significantly increased (*P* < 0.001) (Fig. 3B).

In addition to miR-148a we also analyzed the RNA sequencing data from HCC patients for the expression of miR-148b and miR-152, other miRNA members of miR-148a/152 family; miR-210 and miR-320a that were shown to control TFR1 expression^38,70^. Unlike miR-148a, miR-152 and miR-210 whose expression is significantly lower in tumor compared to the matched normal healthy tissues (Fig. 3B,D,E), the expression of miR-148b is significantly increased in tumor (Fig. 3C). By contrast, no significant changes were observed for miR-320a (Fig. 3F). Moreover, the sequence reads of miR-148a in HCC patients (both in normal and tumor tissues) were significantly higher compared to other members of the miR-148/152 family (Fig. 3G) or miR-210 and miR-320a (Fig. 3H). This may suggest that miR-148a plays the predominant role in controlling TFR1 expression in liver cancer. Importantly, we observed a better survival among HCC patients with high miR-148a (Fig. 3I) and a tendency towards better survival in patients with low TFR1 mRNA expression (Fig. 3J).

**Figure 4:**
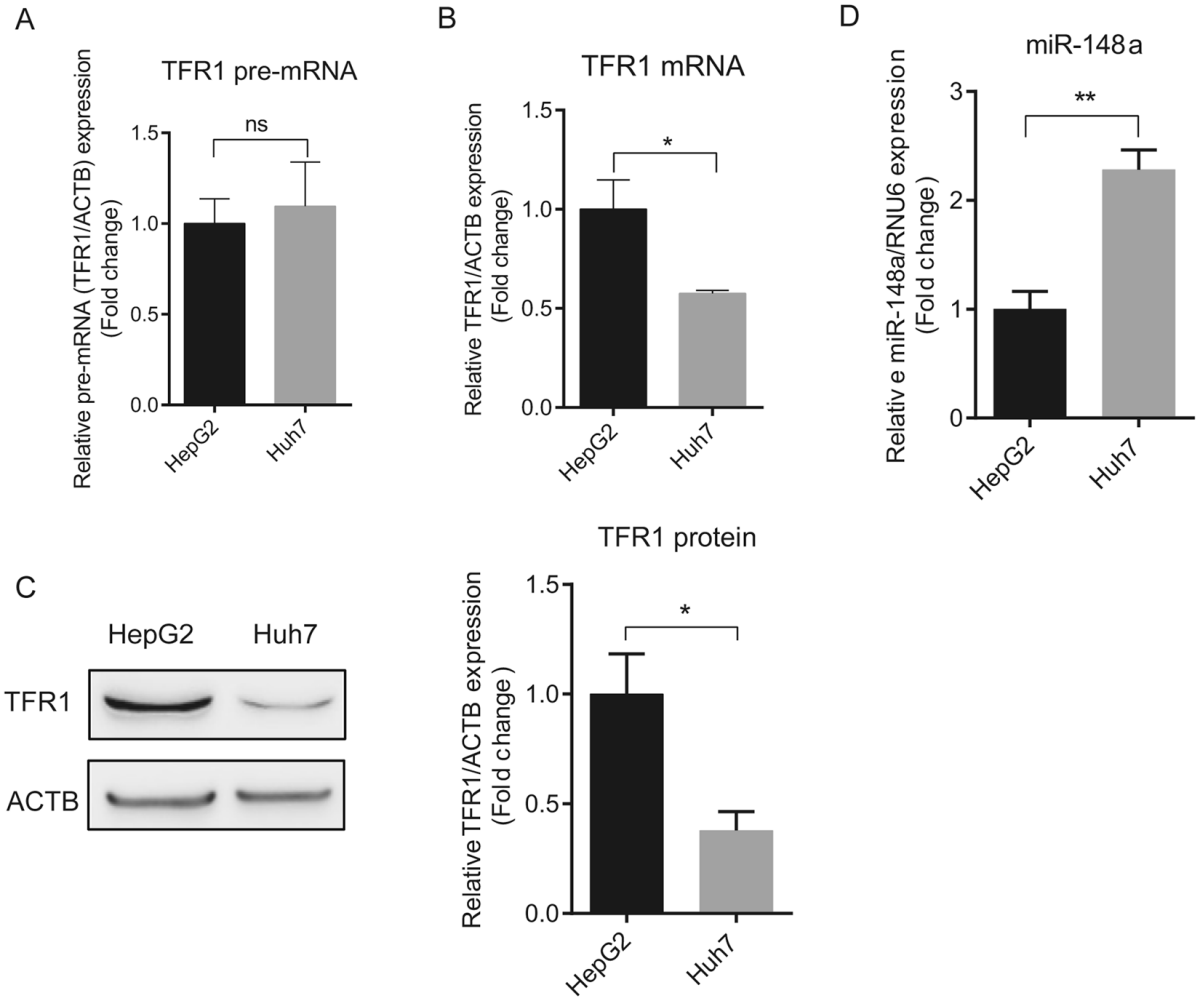
TFR1 expression is negatively correlated to miR-148a levels in HCC cells. Two HCC cell lines were analyzed for the expression of TFR1 pre-mRNA and mRNA by qPCR. (**A**) Differences of TFR1 pre-mRNA levels were similar between HepG2 and Huh7 cells (P = 0.7475), while TFR1 mRNA (**B**) and protein (**C**) levels were significantly different. (**D**) miR-148a expression in the HepG2 and Huh7 cell lines. Experiments were performed in triplicates and repeated at least three times. Q-PCR data were normalized to appropriate reference genes: ACTB (**A**,**B**) or RNU6 (**D**). Representative western blots displayed in C are cropped from Fig. S6. Data are represented as mean ± SEM. ns = no statistical significance, *P < 0.05, **P < 0.01, 2-tailed Student’s t test.

Furthermore, we show a correlation between increased TFR1 mRNA expression and decreased miR-148a expression in human hepatocellular carcinoma (HCC) cell lines (HepG2 and Huh7). While TFR1 pre-mRNA levels were similar between HepG2 and Huh7 cells (*P* = 0.7475) (Fig. 4A), indicating a comparable transcription rate, TFR1 mRNA (Fig. 4B) and protein (Fig. 4C) expression were higher in HepG2 compared to Huh7 cells. By contrast, miR-148a expression was lower in HepG2 compared to Huh7 cells (Fig. 4D), suggesting that TFR1 expression in HepG2 cells may be controlled post-transcriptionally by miR-148a.

### Ectopic miR-148a expression controls TFR1 mRNA expression in HCC cells

**Figure 5:**
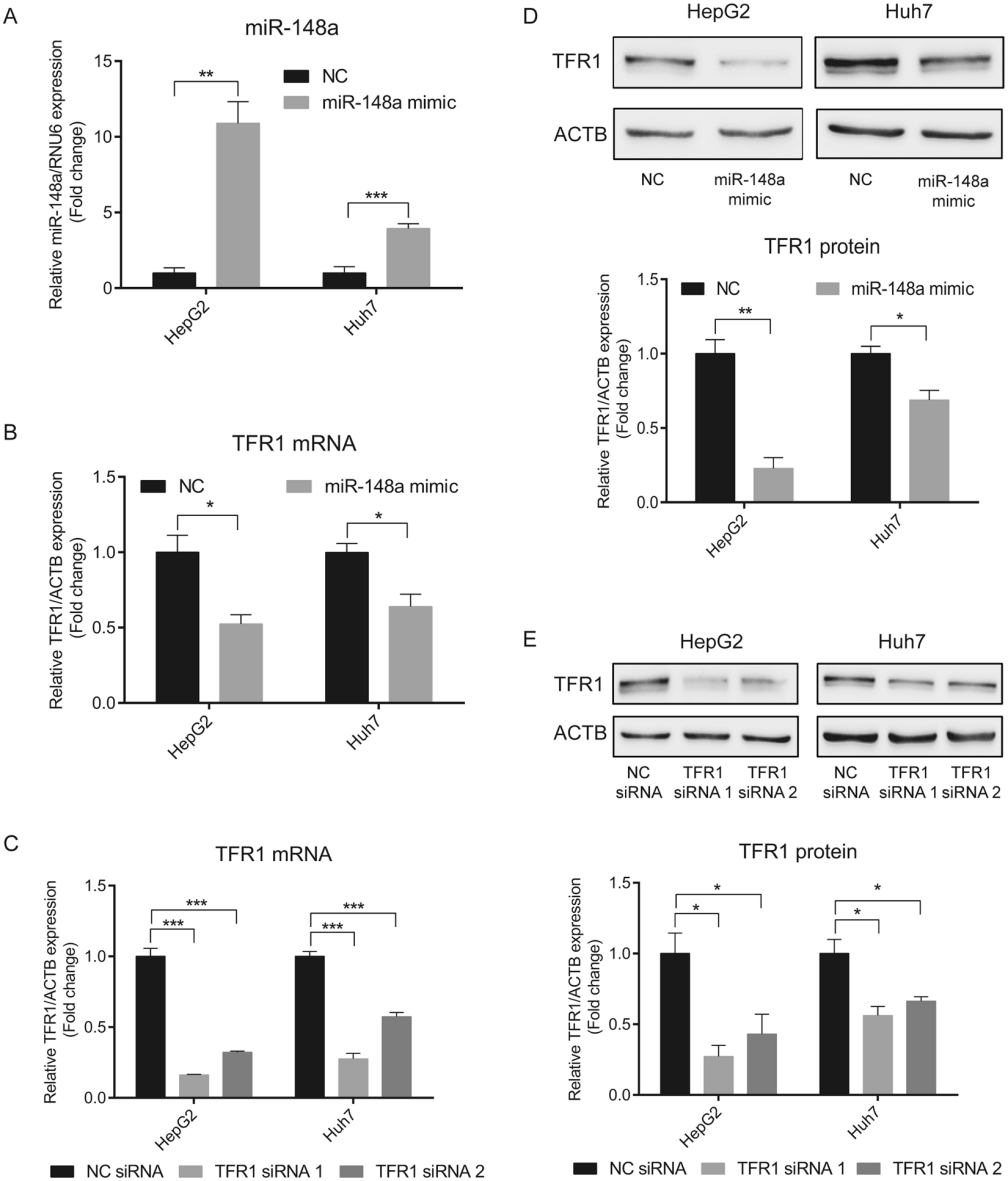
Overexpression of miR-148a decreases TFR1 expression in HCC cells. HepG2 and Huh7 cells were transiently transfected with either a miR-148a mimic, a negative control (NC), specific TFR1 siRNAs (TFR1 siRNA 1 and TFR1 siRNA 2) or a negative control (NC) siRNA. 24 h later, the expression of (**A**) miR-148a and (**B**,**C**) TFR1 mRNA were analyzed by qPCR. TFR1 protein levels were analyzed by western blot analysis following (**D**) miR-148a overexpression or (**E**) TFR1 knock-down. Experiments were repeated at least three times. Q-PCR data were normalized to appropriate reference genes: ACTB (B,C) or RNU6 (**A**). Representative western blots displayed in D and E are cropped from Figs S7 and S8, respectively. Data are represented as mean ± SEM. *P < 0.05, **P < 0.01, ***P < 0.001, 2-tailed Student’s t test.

To investigate whether ectopic expression of miR-148a affects endogenous TFR1 mRNA and protein expression in HCC cell lines, we transfected miR-148a mimics into HepG2 and Huh7 cells. miR-148a levels were significantly elevated (Fig. 5A), while expression of endogenous TFR1 mRNA and protein and of DNMT1 mRNA (positive control) were significantly decreased (Figs 5B,D and S1A, respectively). Expression of RPL19 mRNA (negative control) remained unchanged (Fig. S1B). These data suggest that miR-148a regulates TFR1 expression in HCC cells.

### miR-148a overexpression and TFR1 knockdown decrease HCC cell proliferation

**Figure 6:**
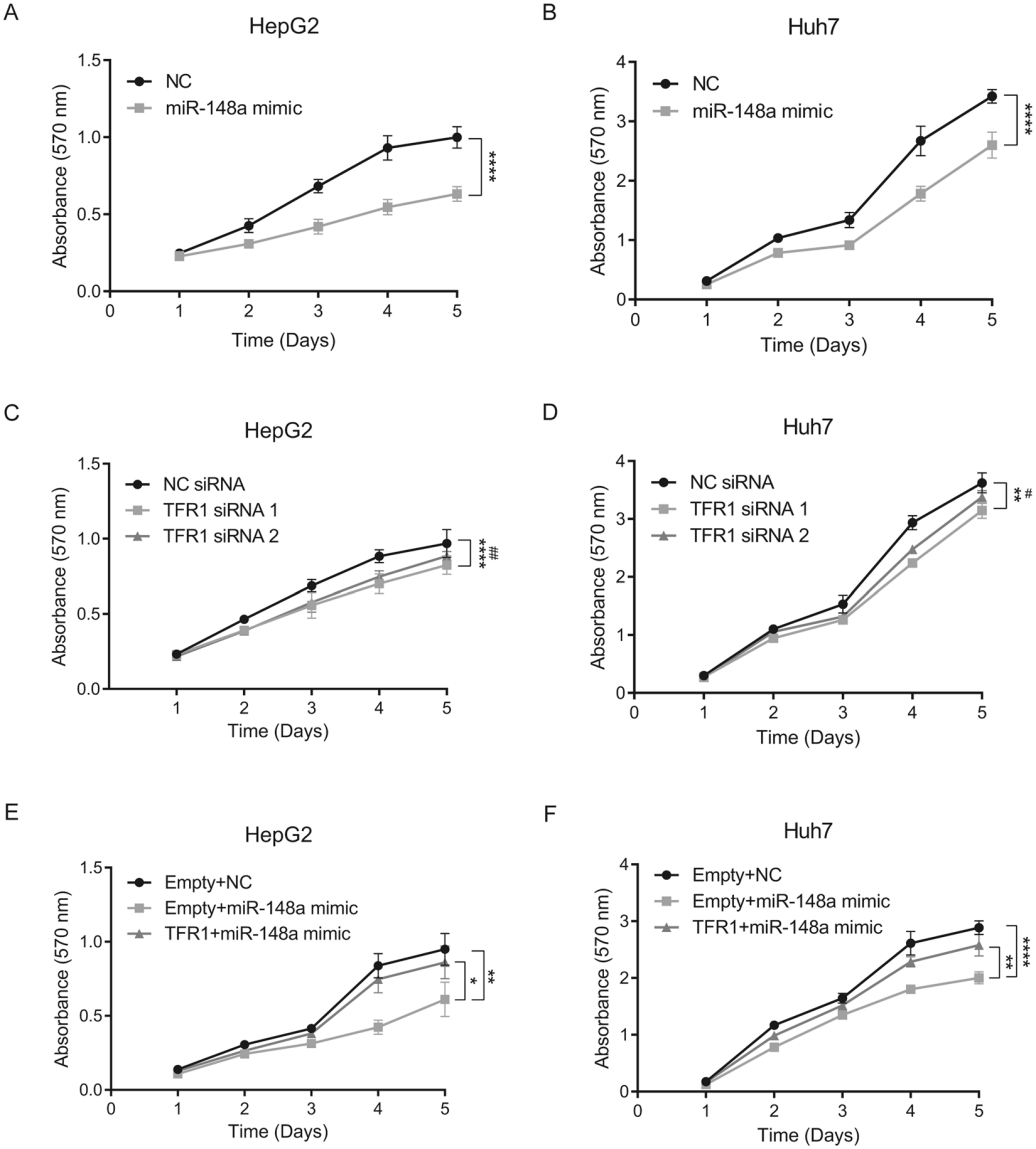
miR-148a overexpression or TFR1 knockdown affects HCC proliferation. HepG2 and Huh7 cells were transiently transfected with either a miR-148a mimic, TFR1 siRNAs, TFR1 + miR-148a mimic, Empty + miR-148a mimic or respective negative controls. 24 h post-transfection, cells were seeded (5000 cells/well) into a 96-well culture plate. The optical density (OD) of (**A**,**C**,**E**) HepG2 and (B, D and F) Huh7 cells treated with crystal violet was measured at 570 nm for up to 5 days. Experiments were performed in replicates and repeated at least three times. Data represented as mean ± SEM. Two-way ANOVA was applied to calculate statistical signification of growth curves (statistical difference between NC siRNA and TFR1 siRNA 2 is represented as #). *P < 0.05, **P < 0.01, ****P < 0.0001, ^#^*P* < 0.05, ^##^*P* < 0.01.

We next investigated the physiological consequences of miR-148a and TFR1 expression in HCC cells. Earlier studies showed that overexpression of miR-148a inhibits cell proliferation of various cancers including breast^5^, bladder^72^ and HCC^21^. Likewise, repression of TFR1 reduces cell proliferation in different cancer entities including breast^73^, esophageal squamous cell^59^, oral squamous cell^61^ and pancreas^62^. Therefore, we hypothesized that miR-148a and TFR1 expression levels may affect cell proliferation. To manipulate miR-148a or TFR1 levels, we transiently transfected HepG2 and Huh7 cells with miR-148a mimics or specific TFR1 siRNAs (TFR1 siRNA 1 and TFR1 siRNA 2), respectively. Following transfection TFR1 mRNA and protein levels were significantly decreased (Fig. 5B–E). The proliferation rate of transiently transfected cells was analyzed by a crystal violet growth curve assay for 5 days. We demonstrate that overexpression of miR-148a in HCC cells suppresses cell proliferation; HepG2 cells (*F*(4,64) = 49.92; *P* < 0.0001) (Fig. 6A) and Huh7 cells (*F*(4,64) = 37.93; *P* < 0.0001) (Fig. 6B). Moreover, knockdown of TFR1 by two different siRNAs in HCC cells significantly decreased cell proliferation; HepG2 cells siRNA 1 (*F*(4,64) = 7.011; *P* < 0.0001) siRNA 2 (*F*(4,64) = 4.346; *P* = 0.0036) (Fig. 6C) and Huh7 cells siRNA 1 (*F*(4,64) = 4.928; *P* = 0.0016) siRNA 2 (*F*(4,64) = 2.727; *P* = 0.0368) (Fig. 6D). To analyze whether TFR1, at least in part, mediates the effects of miR-148a on HCC proliferation, we transiently co-transfected HepG2 and Huh7 cells with pcDNA-TFR1, a mammalian expression plasmid encoding the complete human TFR1 CDS, and the miR-148a mimic (TFR1 + miR-148a mimic). As controls, a pcDNA empty plasmid was co-transfected either with a miR-148a mimic (Empty + miR-148a mimic) or a negative control (Empty + NC). We observed that overexpression of TFR1 in HCC cells (Fig. S2) rescued the suppressive effect of miR-148a; HepG2 cells (*F*(4,64) = 2.805; *P* = 0.0329) (Fig. 6E) and Huh7 cells (*F*(4,64) = 3.783; *P* = 0.0080) (Fig. 6F).

**Figure 7:**
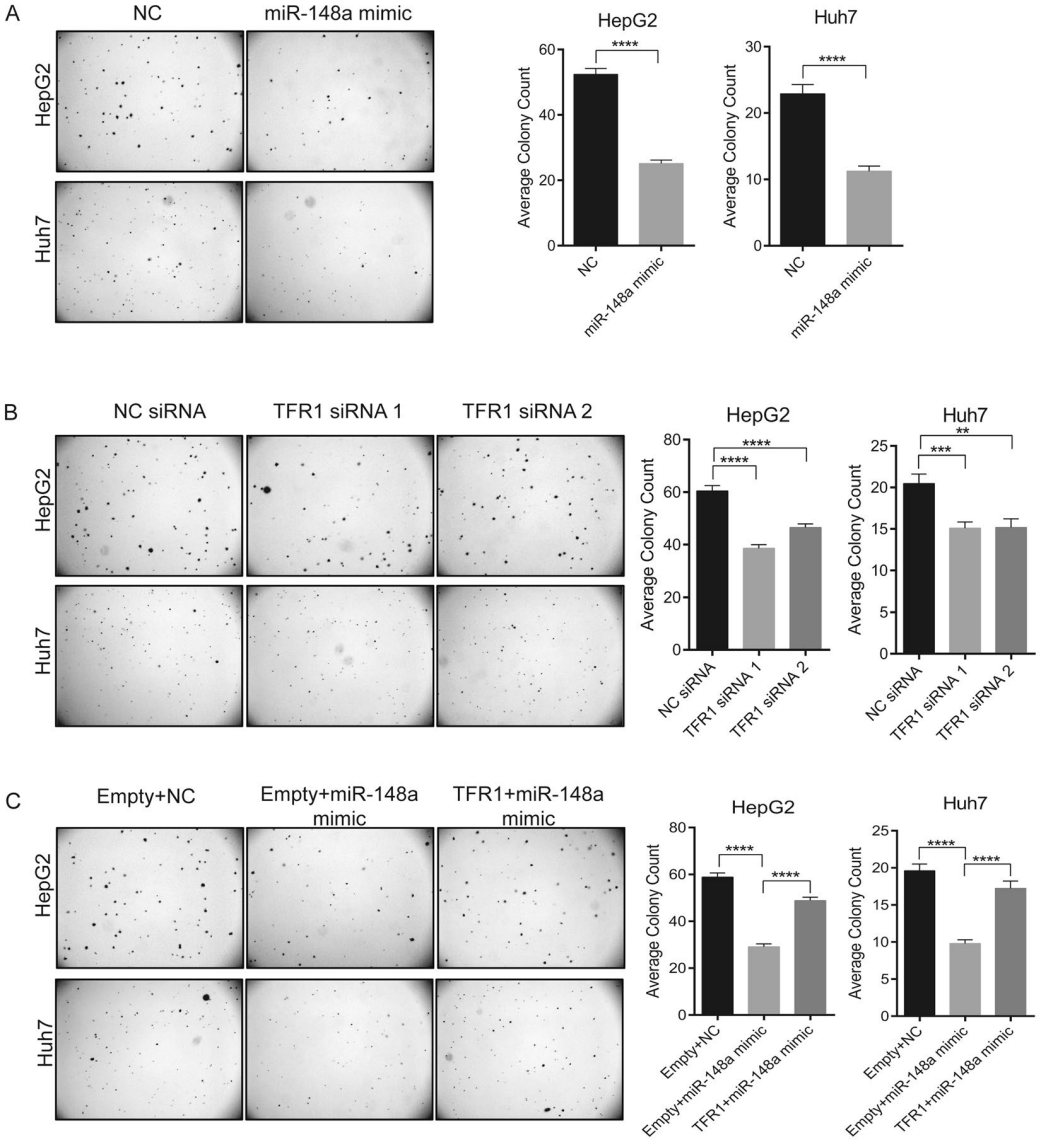
miR-148a overexpression or TFR1 knockdown affects anchorage-independent growth of HCC. HepG2 and Huh7 cells were transiently transfected with either a miR-148a mimic, TFR1 siRNAs, TFR1 + miR-148a mimic, Empty + miR-148a mimic or respective negative controls. 24 h post-transfection, cells were mixed with complete growth medium and 0.3% agarose. The cell mixture (10,000 cells/well) was added above the agarose base in 6-well culture plate. After 10 days of incubation, the colonies were imaged under 0.5X magnification using an Olympus SZX12 microscope and counted using ImageJ v.1.51k. Quantitative data are shown in (**A–C**). Treatments are indicated. *Left*, representative images of colonies formed; *right*, quantitation of colonies. Experiments were repeated at least three times. Data represented as mean ± SEM. **P < 0.01, ***P < 0.001, ****P < 0.0001, 2-tailed Student’s t test.

In addition, we analyzed whether miR-148a and/or TFR1 expression levels impact on the anchorage-independent growth of HCC cells. HepG2 and Huh7 cells were transiently transfected with miR-148a mimic, TFR1 siRNAs, TFR1 + miR-148a mimic, empty + miR-148a mimic or respective negative controls. 24 h post-transfection, cells were mixed with complete growth medium and 0.3% agarose and the mixture was added above the agarose base in 6-well plates (10,000 cells/well). 10 days later, the number of colonies were counted. We show that both the overexpression of miR-148a and the knockdown of TFR1 significantly suppressed the anchorage-independent growth of HCC cells (Fig. 7A,B, respectively). Importantly, overexpression of TFR1 significantly reversed the suppressive effect of miR-148a (Fig. 7C). Altogether, our data show that miR-148a controlled TFR1 regulation is associated to HCC cell proliferation.

## Discussion

We identify TFR1 as a direct target of miR-148a. MiR-148a controls TFR1 expression by binding to two evolutionary conserved MREs at nt 864–870 and nt 1159–1165 within the TFR1 3′UTR (Fig. 1). Additional studies showed miRNA-mediated TFR1 regulation by the hypoxia-induced miR-210^38^ and 12-O-tetradecanoylphobol-13-acetate (TPA)-induced miR-320a^70^, which down regulate the expression of TFR1 by directly targeting the TFR1 3′UTR at nt 2216–2222 and nt 148–154, respectively. A recent study further showed that miR-152, a member of miR-148/152 family directly targets the TFR1 3′UTR and regulates the expression of TFR1 in HCC cells, but the target site was not revealed^60^. These findings suggest that miRNAs play significant roles in regulating TFR1 expression under physiological and pathophysiological conditions, adding an additional layer of expression control^34,49–56^.

We analyzed RNA sequence datasets of HCC patients and observed a significant negative correlation between mRNA expression levels of TFR1 mRNA and miRNA members of the miR-148/152 family. Unexpectedly, for miR-148b a significant positive correlation was observed (Figs 3A, S3A and B). Because TFR1 mRNA levels are low in tumor samples when miR-148a and miR-152 is increased these seem to show a dominant effect over miR-148b. In addition to miR-148a/152 family members we also screened the RNA sequencing data from HCC patients for the expression of miR-210 and miR-320a. Similar to miR-148b, we observed a significant positive correlation between expression levels of miR-210 and TFR1 mRNA (Fig. S3C). Whereas, no significant change was observed for miR-320a (Fig. S3D).

In addition, we applied mimiRNA, a miRNA expression profiler^64^ to analyze mRNA expression of TFR1 and miRNAs of the miR-148/152 family, miR-210 or miR-320a across multiple human tissues and cell types. This analysis revealed a highly significant negative correlation between the expression of TFR1 and miR-148a (*r* = −0.435, *P* = 0.001) (Fig. S4A) and between TFR1 and miR-210 (*r* = −0.284, *P* = 0.042) (Fig. S5A). By contrast, no significant correlations were detectable for miR-148b, miR-152 and miR-320 (Figs S4B, C and S5B, respectively). These results suggest that miR-148a and miR-210 may control TFR1 expression levels in most physiological and pathophysiological conditions.

HCC patients with low TFR1 expression show a trend towards better survival compared to individuals with high TFR1 expression (Fig. 3J). However, the difference was not statistically significant when applying Mantel-cox test (*P* = 0.0672) which gives equal weight to all time points of death analyzed. Interestingly when applying Gehan-Breslow-Wilcoxon test, which gives more weight to those deaths occurring at early time points, the difference in survival was statistically significant (*P* = 0.0041). This suggests that high TFR1 levels and thus an elevated potential for iron uptake at early time points of HCC may confer a higher risk. It is of note that patients with glioblastoma also show better survival with low TFR1 expression^74^.

It is important to note that expression of miR-148a is repressed by the oncogenic transcription factor c-Myc, whereby c-Myc is directly targeted by miR-148a forming a Myc-miR-148a feedback loop^18^. Here we show that TFR1 is a direct target of miR-148a, but TFR1 is also regulated by c-Myc^53^. Likewise, miR-320a directly targets both, c-Myc^75^ and TFR1^70^. These data suggest that the mRNAs of TFR1 and c-Myc may function as competing endogenous RNAs (ceRNAs)^76^, thus increasing the complexity how TFR1 expression is controlled.

We demonstrate that increased miR-148a levels downregulate the expression of endogenous TFR1 mRNA and protein in HCC cell lines (HepG2 and Huh7) (Fig. 5B,D) and that suppression of TFR1 expression directly or mediated by miR-148a overexpression decreases HCC cell proliferation (Figs 6,7). How does decreased TFR1 expression relate to cancer cell proliferation? Earlier investigations demonstrated that suppression of TFR1 in cancer cells decreases transferrin uptake and the intracellular labile iron pool (LIP)^55,60,61,73^, thereby reducing intracellular iron availability. Iron is essential to regulate proteins that control the cell cycle^29,30,42^ and cell proliferation signaling pathways^31–33^. This may explain why low TFR1 levels in HCC are associated with an improved prognosis.

## Conclusion

We provide functional evidence that increased TFR1 levels in liver cancer can be caused by reduced miR-148a expression, which is observed in various cancer subtypes. Suppression of TFR1 expression reduces proliferation of HCC cells due to decreased iron uptake and cellular iron availability and may explain improved survival of HCC patients. Thus, post-transcriptional regulation of TFR1 by miR-148a may serve as a potential anti-cancer therapy.

## Electronic supplementary material

Supplementary Information

## Data Availability

The datasets analyzed during the current study are available in the UCSC Xena browser repository, http://xena.ucsc.edu/.
