# Supplementary material for: miR-148a regulates expression of the transferrin receptor 1 in hepatocellular carcinoma
Source: Sci Rep. 2019 Feb 6;9:1518. doi: 10.1038/s41598-018-35947-7 (PMC6365501; doi:10.1038/s41598-018-35947-7)
Supplement: Supplementary file 1 — Supplementary Information [file 41598_2018_35947_MOESM1_ESM.pdf]

Supplementary Information

miR-148a regulates expression of the transferrin receptor 1 in hepatocellular carcinoma

Kamesh R. Babu and Martina U. Muckenthaler

Name	5' Sequence 3'	Tm (°C)	Product size (bp)
hs-ACTB_F	TTCCGCTGCCCTGAGGCACTCT	65.3	
hs-ACTB_R	TCTGCTGGAAGGTGGACAGCGA	62.8	301
hs-pre-ACTB_F	CTATTCTCGCAGCTCACCATG	55.6	
hs-pre-ACTB_R	CAGCTCCCCTACCTGGTG	57.5	153
hs-pre-TFR1_F	TTTCCGCAACACAGTTTGGT	55.6	
hs-pre-TFR1_R	TGGTTTTGTGACATTGGCCT	55.3	155
hs-TFR1_F	CTGCTTTCCCTTTCCTTGCATATT	55.9	
hs-TFR1_R	GCTCGTGCCACTTTGTTCAACT	58.4	148
hs-DNMT1_F	CTTCACCTAGCCCCAGGATT	56.5	
hs-DNMT1_F	ACGTCTCTTCTCATCCTGGTC	55.9	164
hs-pre-RPL19_F	GTAGTGGCCCGTTCCTAACT	56.5	
hs-pre-RPL19_R	GTGTGCTTACATGTGGCGAT	55.8	165
hs-RPL19_F	TCGCCTCTAGTGTCCTCCG	58.4	
hs-RPL19_R	GCGGGCCAAGGTGTTTTTC	57.7	182
*hs-RNU6_F	GTGCTCGCTTCGGCAGCACATATAC	61.9	N/A
*hs-miR-148a-3p_F	TCAGTGCACTACAGAACTTTGTG	55.2	N/A

Table S1: Primers used for qPCR analysis of mRNA and miRNA transcripts. Forward primers used for miRNA qPCR analysis are marked by *. A proprietary universal reverse primer was provided in the miScript SYBR-Green PCR kit. PCR products of miRNA qPCR are of unknown size, N/A= Not Available.

**Name**	**5' Sequence 3'**
hs-TFR1-3'UTR_F	GA**GAGCTC**CTCTGGTGACGTTTGGGACA
hs-TFR1-3'UTR_R	GA**GCTAGC**AGTGGCAGAAACAGTGGATG
hs-DNMT1-3'UTR_F	GA**GAGCTC**GGAGGAAGCTGCTAAGGACT
hs-DNMT1-3'UTR_R	GA**GCTAGC**CCAGAAGTCCCGTGCAAATC
hs-TFR1-CDS_F	GA**GGATCC**ATGATGGATCAAGCTAGATCAGC
hs-TFR1-CDS_R	CC**TCTAGA**TTAAAACTCATTGTCAATGTCCCAAAC
hs-RPL19_F	[Phos]CAACCTCCCACTTTGTCTGTACATACTGGCCTCTGTGATTACATAGA TCAGCCATTAAAATAAAACAAGCCTTAATCTGCG
hs-RPL19_R	[Phos]CTAGCGCAGATTAAGGCTTGTTTTATTTTAATGGCTGATCTATGTAA TCACAGAGGCCAGTATGTACAGACAAAGTGGGAGGTTGAGCT
hs-miR-148a-3p(+)_F	[Phos]CACAAAGTTCTGTAGTGCACTGAG
hs-miR-148a-3p(+)_R	[Phos]CTAGCTCAGTGCACTACAGAACTTTGTGAGCT
hs-miR-148a-3p(-)_F	[Phos]CTCAGTGCACTACAGAACTTTGTG
hs-miR-148a-3p(-)_R	[Phos]CTAGCACAAAGTTCTGTAGTGCACTGAGAGCT

Table S2: Primers used for the amplification of 3'-UTR and CDS sequences. Restriction site on the primer sequences are boldfaced. Sequences with 5'phosphate represented as [Phos] were annealed using oligo annealing buffer (Promega) followed by direct ligating into the pmirGLO vector.

Name	**5' Sequence 3'**
hs-TFR1-MT1_F	GTCACTGATAAATAAACAATAAATATCT**GTCA**GCCAAAAGGACAGAAAG CTCTCCCCTA
hs-TFR1-MT1_R	TAGGGGAGAGCTTTCTGTCCTTTTGGC**TGAC**AGATATTTATTGTTTATTTA TCAGTGAC
hs-TFR1-MT2_F	GAAAACCAGCATTCTTATCTGGT**GTCA**GCTCGCTTCTTAGCAACCCCTAA
hs-TFR1-MT2_R	TTAGGGGTTGCTAAGAAGCGAGC**TGAC**ACCAGATAAGAATGCTGGTTTTC
hs-DNMT1-MT_F	ATTGACATGTTAAAAACACAACAT**GTCA**GCATGTTGGGGATTCCTGGTGC C
hs-DNMT1-MT_R	GGCACCAGGAATCCCCAACATGC**TGAC**ATGTTGTGTTTTTAACATGTCAA T

Table S3: Primers used to mutagenize the miR-148a-3p response elements. Altered nucleotide sequences are boldfaced.

Figure S1

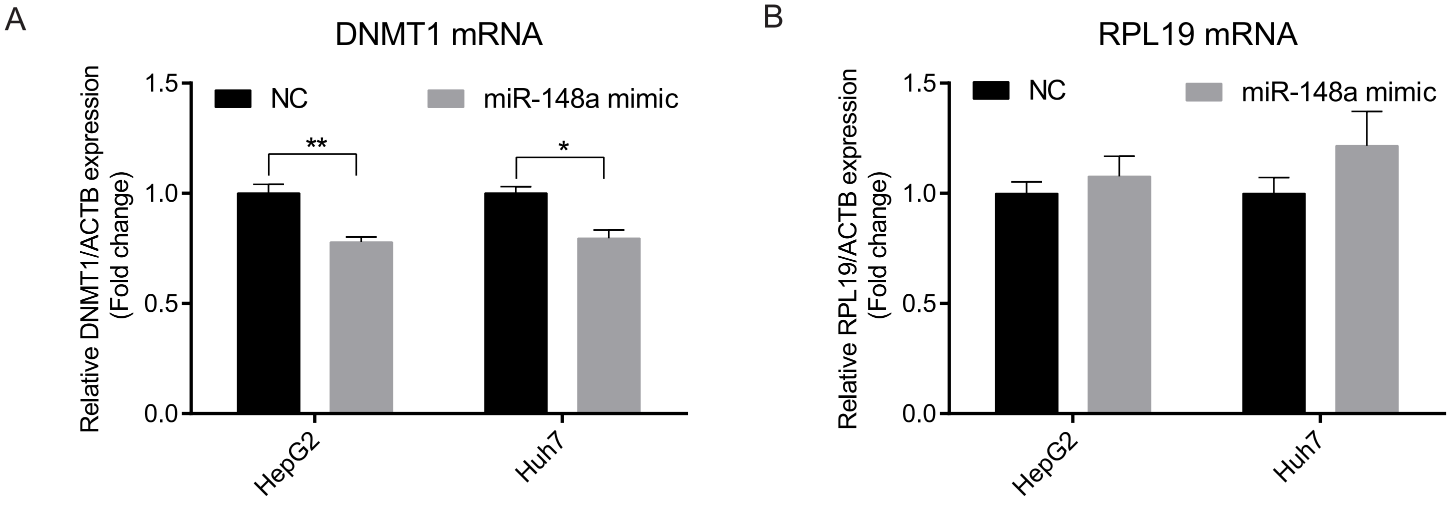

Figure S1. HepG2 and Huh7 cells were transfected with 50 nM of miR-148a mimic or negative control (NC). At 24 h post-transfection (A) DNMT1 mRNA and (B) RPL19 mRNA levels were analyzed by qPCR. Experiments were performed in triplicates and repeated at least three times. Data are represented as mean ± SEM, and the values from NC were set to 1, **P* < 0.05, ***P* < 0.01, 2-tailed student's t test.

Figure S2

A

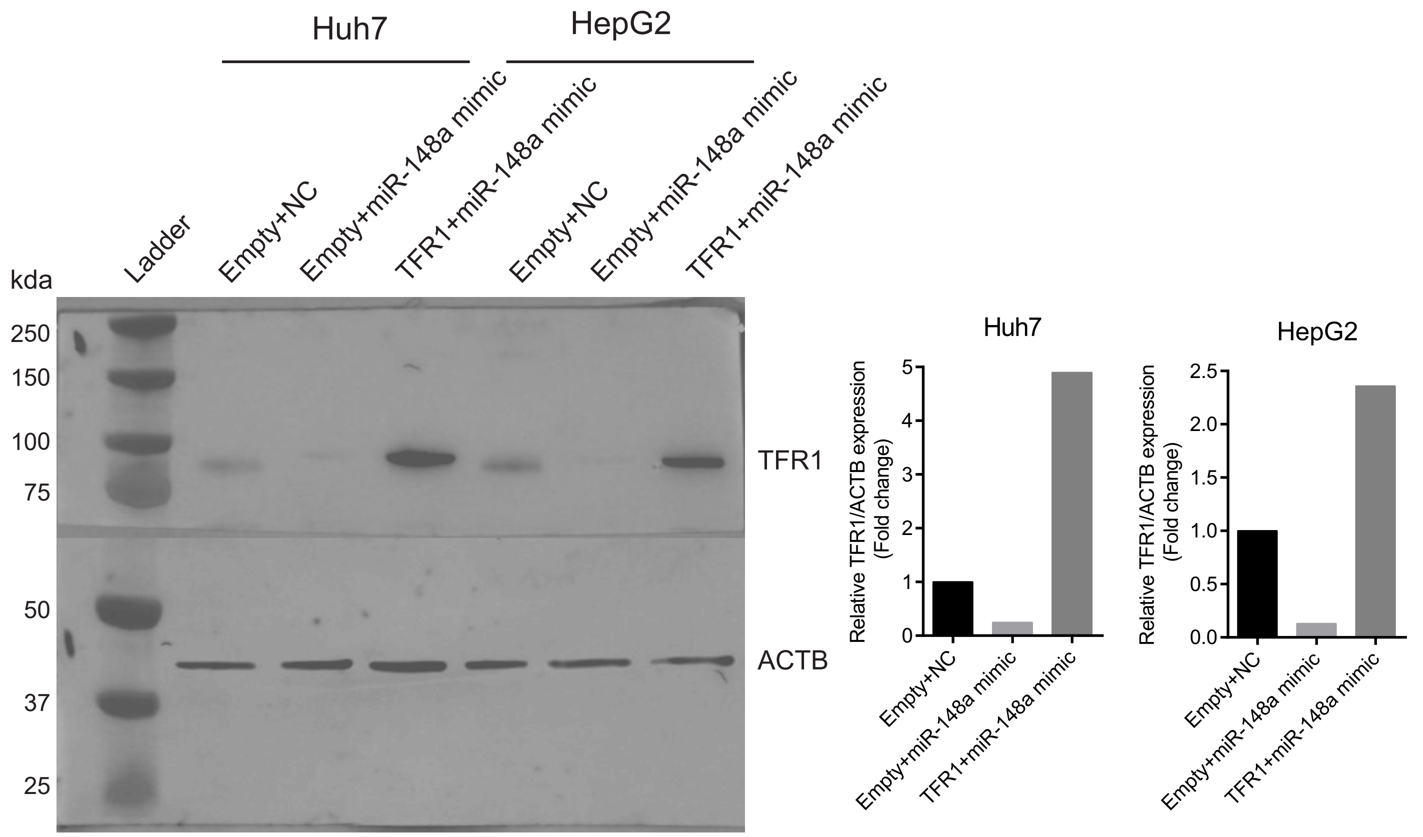

Figure S2. Western blot analyses of TFR1 protein in HepG2 and Huh7 cells from 24 hrs post-transfection of pcDNA empty plasmid and negative control (Empty+NC); pcDNA empty plasmid and miR-148a (Empty+miR-148a mimic); or pcDNA-TFR1 plasmid and miR-148a mimic (TFR1+miR-148a mimic). For semi-quantitative analysis TFR1 signals were normalized with ACTB signals.

Figure S3

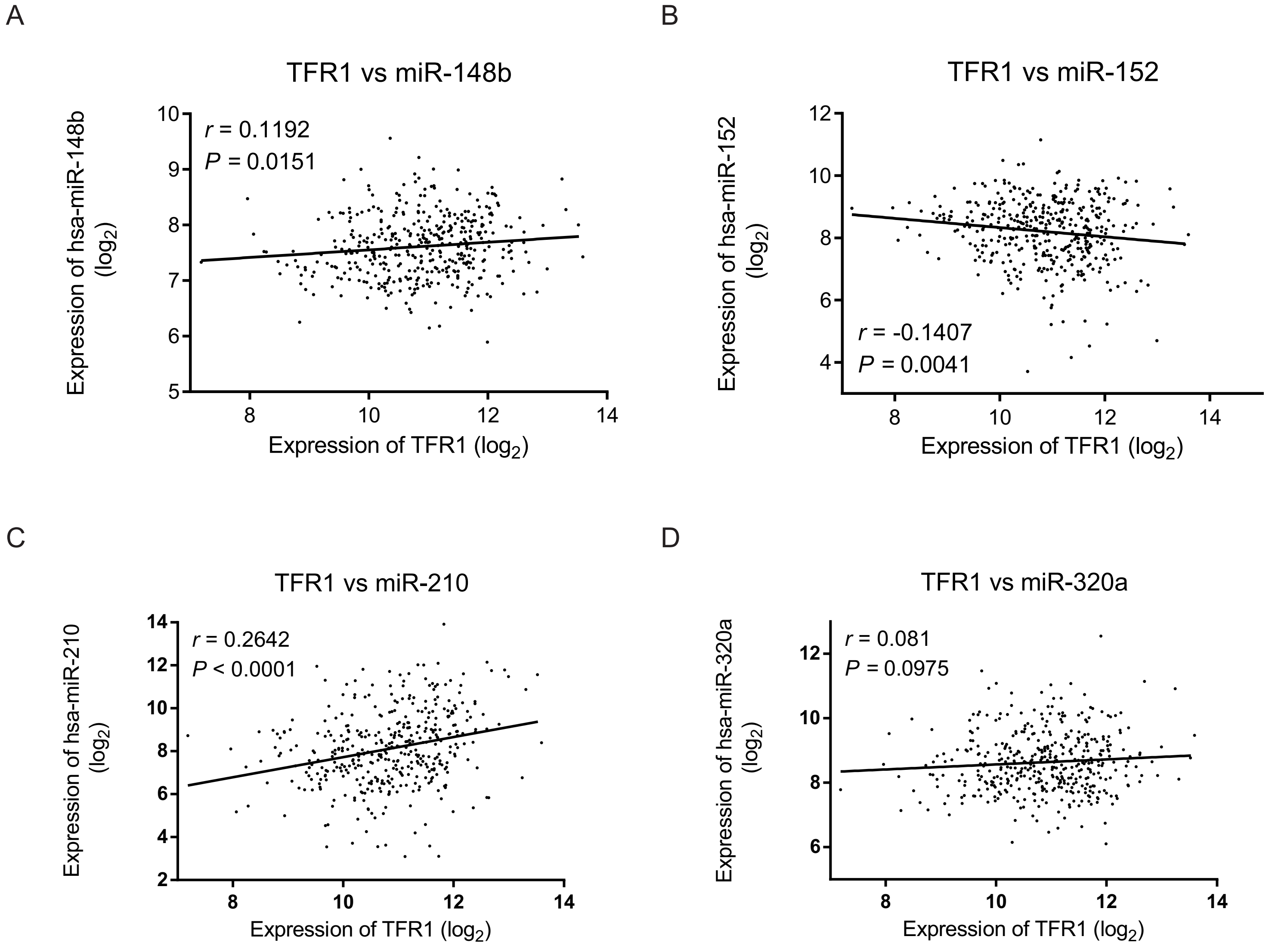

Figure S3. Scatter plots illustrating correlations of TFR1 mRNA levels to (A) miR-148b, (B) miR-152, (C) miR-210 and (D) miR-320a in TCGA datasets of liver hepatocellular carcinoma (LIHC). Pearsons's correlation coefficient was applied.

Figure S4

A

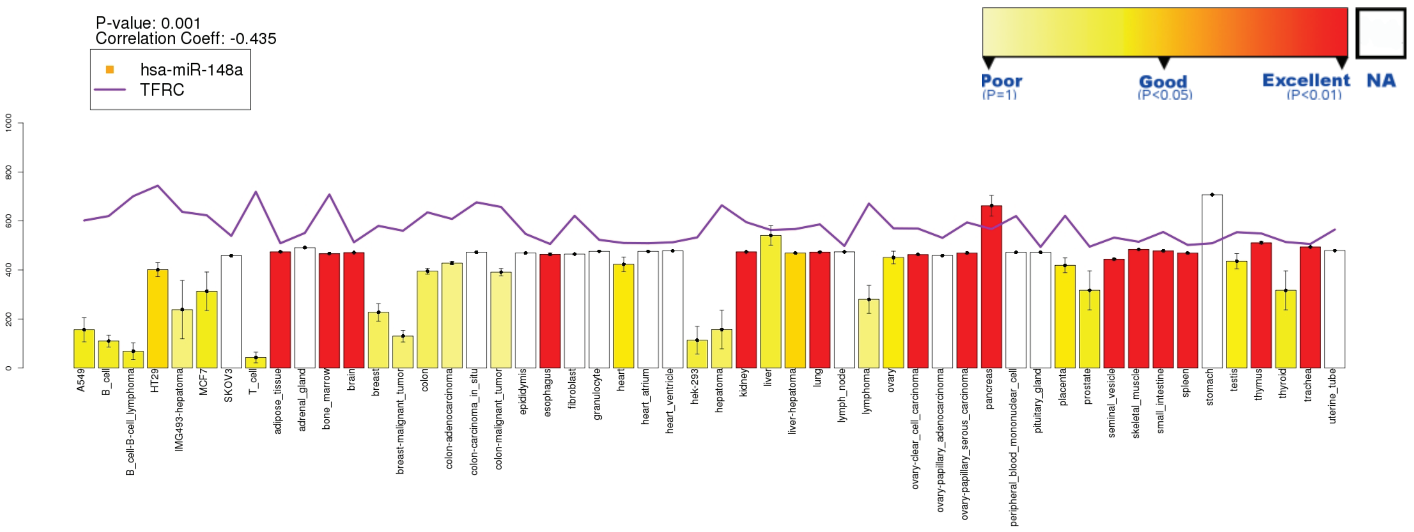

B

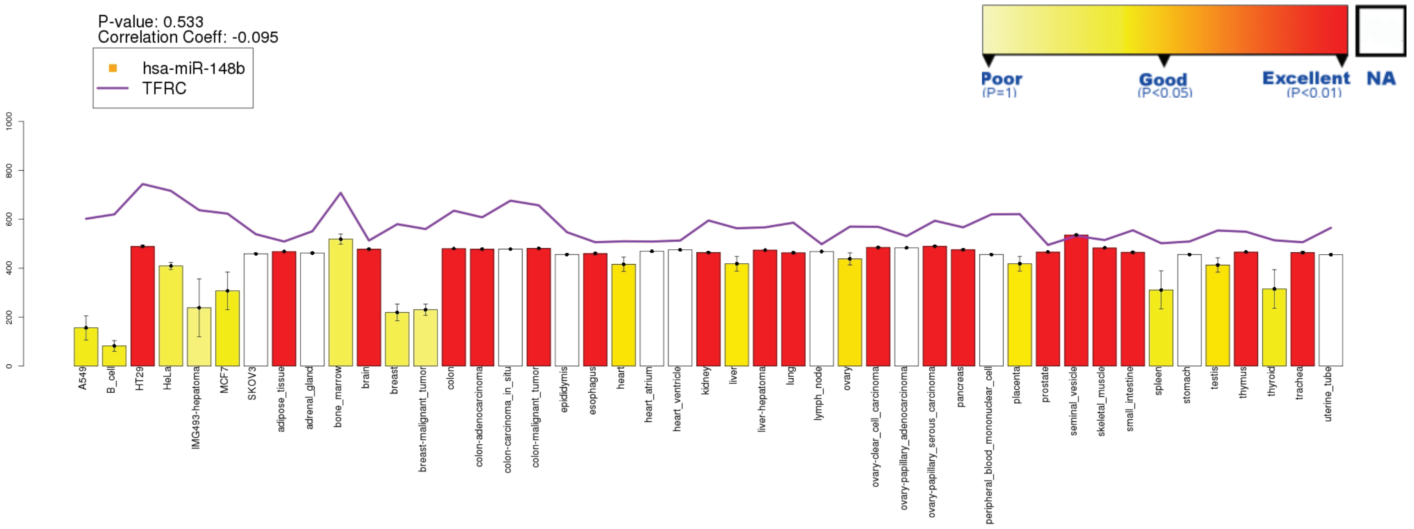

C

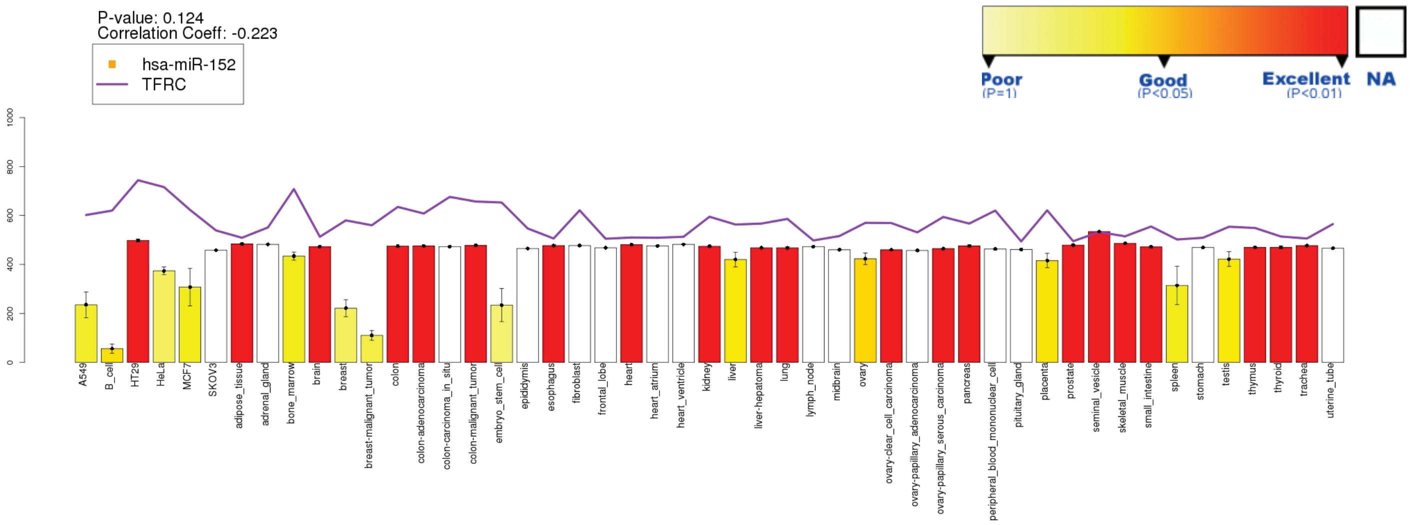

Figure S4. Correlation of TFR1 (represented here as TFRC) mRNA levels to (A) miR-148a, (B) miR-148b and (C) miR-152 across multiple human tissues and cell types. Pearson's correlation coefficient was used.

Figure S5

A

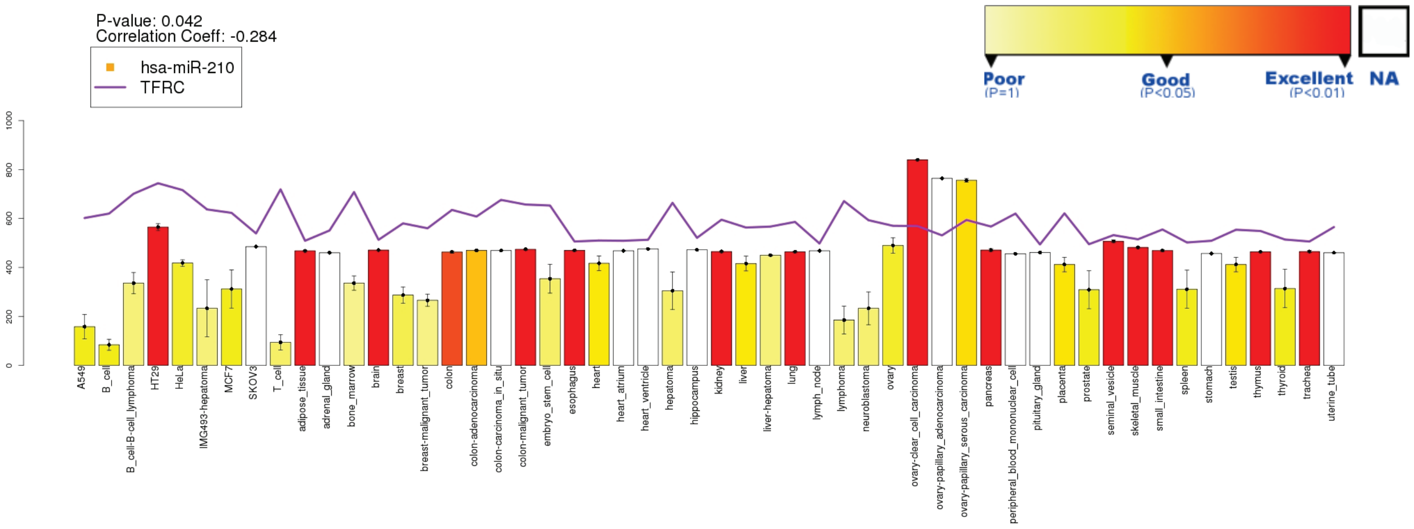

B

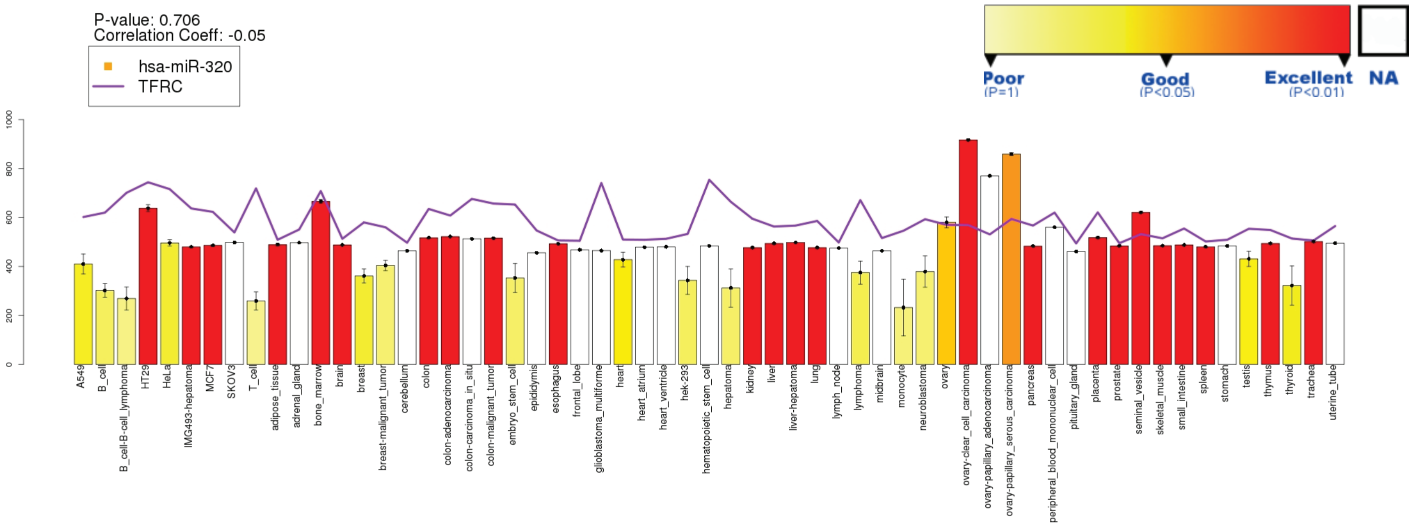

Figure S5. Correlation of TFR1(represented here as TFRC) mRNA levels to (A) miR-210 and (B) miR-320a across multiple human tissues and cell types. Pearson's correlation coefficient was applied.

Figure S6

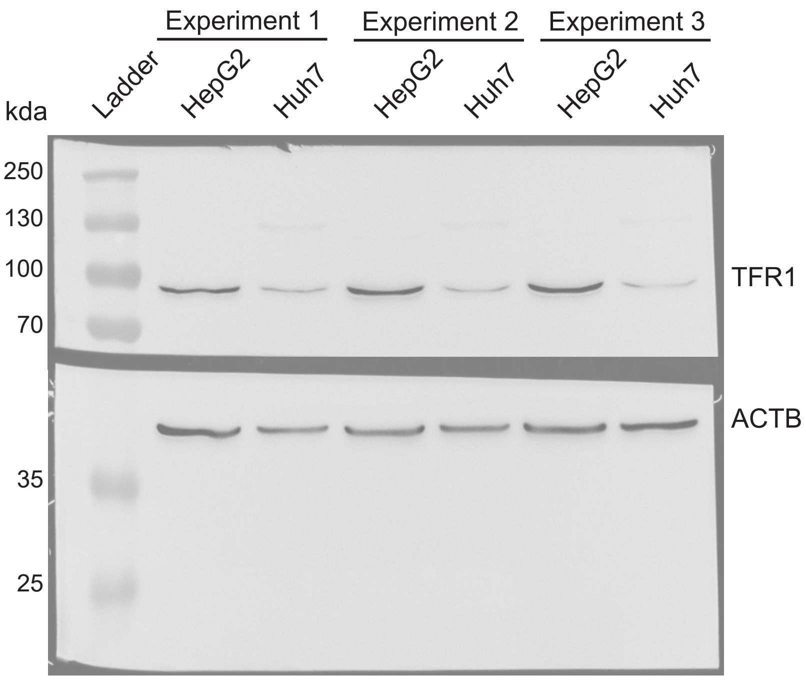

Figure S6.Western blot analyses of TFR1 protein in HepG2 and Huh7 cells.
For semi-quantitative analysis TFR1 protein signals were normalized with ACTB signals.

Figure S7

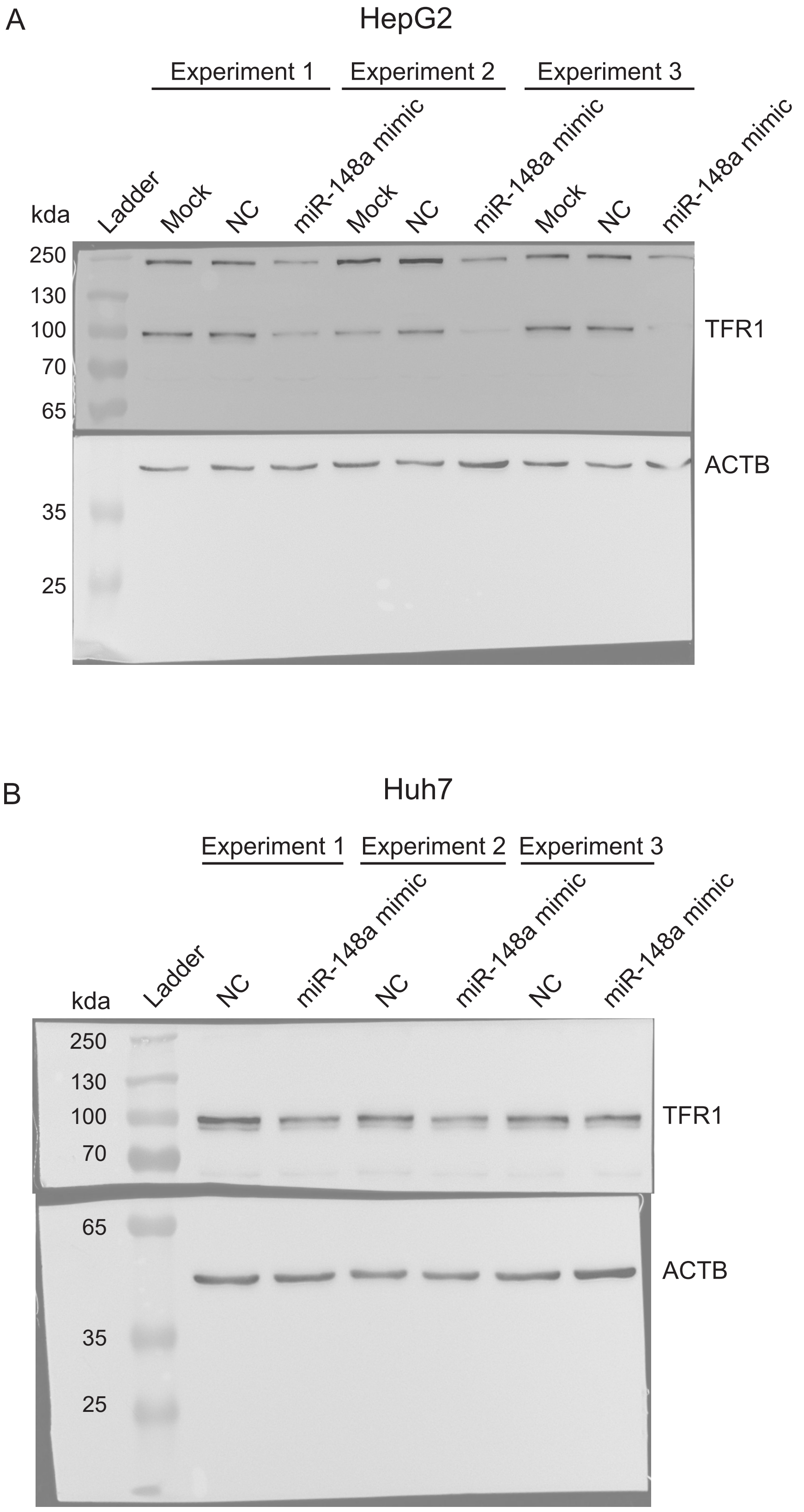

Figure S7. Western blot analyses of TFR1 protein in (A) HepG2 and (B) Huh7 cells from 24 hrs post-transfection of miR-148a mimic or negative control (NC). For semi-quantitative analysis TFR1 signals were normalized with ACTB signals.

Figure S8

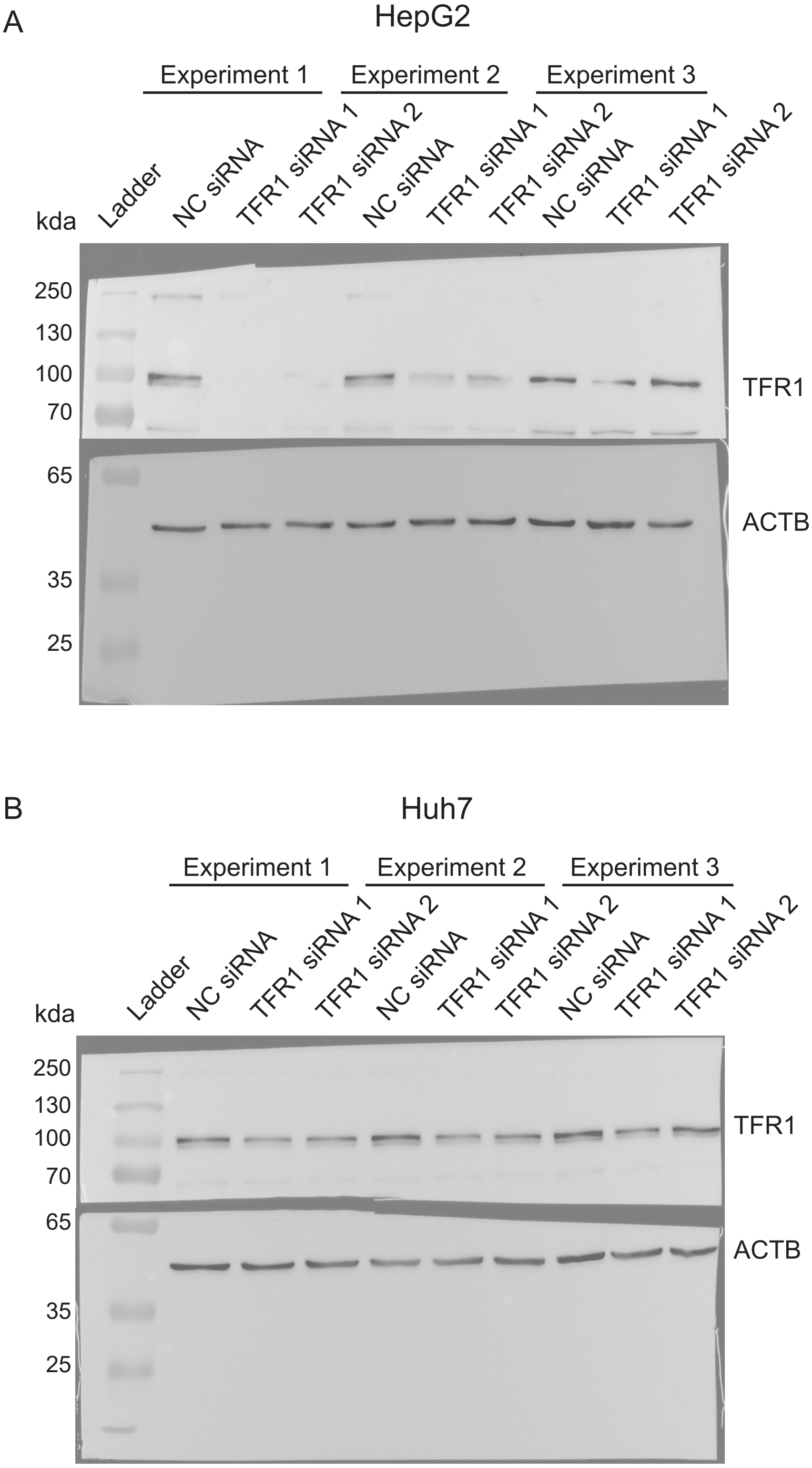

Figure S8. Western blot analyses of TFR1 protein in (A) HepG2 and (B) Huh7 cells from 24 hrs post-transfection of TFR1 siRNA 1, TFR1 siRNA 2 or negative control (NC siRNA).
For semi-quantitative analysis TFR1 signals were normalized with ACTB signals.
